# Diagnostic test accuracy of diabetic retinopathy screening by physician graders using a hand-held non-mydriatic retinal camera at a tertiary level medical clinic

**DOI:** 10.1186/s12886-019-1092-3

**Published:** 2019-04-08

**Authors:** Mapa Mudiyanselage Prabhath Nishantha Piyasena, Jennifer L. Y. Yip, David MacLeod, Min Kim, Venkata S. Murthy Gudlavalleti

**Affiliations:** 1 Clinical Research Department, International Centre for Eye Health, London School of Hygiene and Tropical Medicine, Keppel Street, London, WC1E 7HT UK; 2 Public Health Ophthalmology, International Centre for Eye Health, Clinical Research Department, London School of Hygiene and Tropical Medicine, Keppel Street, London, WC1E 7HT UK; 3 Tropical Epidemiology Group, Department of Infectious Disease Epidemiology, London School of Hygiene and Tropical Medicine, Keppel Street, London, WC1E 7HT UK; 4 Public Health for Eye Care and Disability, International Centre for Eye Health, Clinical Research Department, London School of Hygiene and Tropical Medicine, Keppel Street, London, WC1E 7HT UK

**Keywords:** Diabetes, Diabetic retinopathy, Diagnostic accuracy, Digital imaging, Screening

## Abstract

**Background:**

The evidence on diagnostic test accuracy (DTA) of diabetic retinopathy (DR) screening utilising photographic studies by non-ophthalmologist personnel in low and middle-income country (LMIC) settings is scarce. We aimed to assess DTA of DR screening using a nonmydriatic hand-held digital camera by trained general physicians in a non-ophthalmic setting.

**Methods:**

This study is a validation of a screening intervention. We selected 700 people with diabetes (PwDM) > 18 years of age, not previously screened or treated for DR, presenting at a tertiary medical clinic in Sri Lanka. Two-field retinal imaging was used to capture fundus images before and after pupil dilatation, using a hand-held non-mydriatic (Visuscout 100®-Germany) digital retinal camera. The images were captured and graded by two trained, masked independent physician graders. The DTA of different levels of DR was assessed comparing physician’s grading with a retinologist’s clinical examination by mydriatic bio-microscopy, according to a locally adopted guideline.

**Results:**

Seven hundred eligible PwDM were screened by physician graders. The mean age of participants was 60.8 years (SD ±10.08) and mean duration of DM was 9.9 years (SD ±8.09). Ungradable image proportion in non-mydriatic imaging was 43.4% (either eye-31.3%, both eyes 12.1%). This decreased to 12.8% (either eye-11.6%, both eyes-1.2%) following pupil dilatation. In comparison to detection of any level of DR, a referable level DR (moderate non-proliferative DR and levels above) showed a higher level of DTA. The sensitivity of the defined referable DR was 88.7% (95% CI 81.7–93.8%) for grader 1 (positive predictive value [PPV] 59.1%) and 92.5% (95% CI 86.4–96.5%) for grader 2 (PPV 68%), using mydriatic imaging, after including ungradable images as screen positives. The specificity was 94.9% (95% CI 93.6–96.0%) for grader 1 (negative predictive value [NPV] 99%) and 96.4% (95% CI 95.3–97.3%) for grader 2 (NPV 99.4%).

**Conclusions:**

The Physicians grading of images from a digital hand-held non-mydriatic camera at a medical clinic, with dilatation of pupil of those who have ungradable images, provides a valid modality to identify referable level of DR. This could be a feasible alternative modality to the existing opportunistic screening to improve the access and coverage.

**Trial registration:**

Current Controlled Trials ISRCTN47559703. Date of Registration 18th March 2019, Retrospectively registered.

**Electronic supplementary material:**

The online version of this article (10.1186/s12886-019-1092-3) contains supplementary material, which is available to authorized users.

## Background

Diabetic retinopathy (DR) is a common complication of diabetes mellitus (DM), leading to sight loss if not detected and treated in time [1]. The International Diabetes Federation (IDF) estimated that cases of DM will increase to 629 million by 2045, with a significant burden (80%) in low and middle income countries (LMIC) [2]. Systematic DR screening (DRS) is a challenge in many of the LMICs due to limited resources [3]. The St Vincent declaration stated that all nations should make efforts to reduce DM related complications, including DR blindness [4]. These recommendations were followed by most of the high-income countries (HICs). The LMICs would also be able to achieve this aim with the adaptation and use of existing technologies according to the local contextual requirements.

The most common method of detecting DR in LMICs is direct ophthalmoscopy and slit-lamp bio-microscopy. The direct ophthalmoscopy has a low sensitivity and specificity even at the hands of experienced eye care specialists [5]. The mydriatic bio-microscopic examination by an ophthalmologist is not practical in these countries due to the low number of ophthalmologists and eye clinics which are already over-subscribed with more common blinding conditions such as cataract [6]. In these circumstances, DR is detected through opportunistic case detection only. Insufficient capacity and lack of screening infrastructure hampers efforts to implement DRS programs (DRSP) in these settings, and there is a lack of evidence of what works in LMICs [7–9].

Different models of DRS have been implemented in many parts of the world. In resource poor LMICs development of a DRS model is complex [10]. The lack of trained human resources and infrastructure has outstripped the capacity to deliver systematic DRS in low income settings [11]. There are also poor recording systems to identify the people with DM (PwDM). Therefore, a comprehensive population-based DRSP may not be feasible in LMICs in the near future [8, 10]. DRS also requires appropriate integration into routine care for sustainability [10]. It was shown that public health integration of DRS is a feasible strategy to control avoidable blindness [12]. As such, one feasible model of systematic DRS in LMICs could be screening of PwDM when they attend for routine medical care. This can provide a participant list of PwDM, who can be offered screening at regular intervals. Integrated DRS at medical care clinics can also facilitate risk stratification and prioritisation of referrals to busy eye clinics. In these circumstances, a key consideration would be the availability of skilled human resources facilitating task shifting and sharing and efficient, cost effective and valid technology for DRS.

Retinal fundus photography is the most common DRS method used globally [13] and digital systems are mostly preferred [14]. Conventional desk-top digital cameras require significant physical space, skilled photographers and large image storage devices which incur high capital investment but are cost effective [15]. Hand-held digital cameras are portable, require less space, minimum power consumption and less skills and training [16]. Non-mydriatic retinal imaging is more popular considering the convenience for both service user and provider due to absence of procedures such as pupil dilatation [17]. However, this may have an impact on image gradability and screening coverage [18].

Hand held retinal cameras use for DRS in various settings and outcomes mainly depend on the image quality. Yogesan et al., (2000) reported that images captured by a hand-held camera were not suitable for tele-screening due to poor quality (only 24% in good quality). However in this study sample size was very low (*n* = 25 participants, 49 eyes) [19]. A study conducted in France, concluded that hand-held retinal imaging system was less efficient with poor image quality. However, in this study the photographer had undergone training only on 10 patients before the study, which is a highly inadequate for a hand-held camera [20]. In contrast, A study conducted in China reported that 63% of the images were in excellent quality, however the age of the participants was started as low as 9 years (age range 9–84 years) [21]. A review by Cuadros et al., (2017) concluded that hand-held cameras are practically convenient but do not provide sufficient image quality [22]. Therefore, quality of the images is a major concern in hand-held devices, though they are easy to use.

To the authors’ knowledge there is no evidence on DRS using digital retinal imaging from Sri Lanka. A situational analysis conducted in the Western province showed a large gap in DRS services delivery compared to the estimated need [23]. This study aims to demonstrate the functional and technical feasibility of using a hand-held non-mydriatic digital camera in a LMIC non-ophthalmic setting. We assessed the DTA of DR detection by general physicians using this method compared to the local clinical reference standard of mydriatic indirect ophthalmoscopy and bio-microscopic examination by a retinologist.

## Methods

Ethics approval was obtained from both ethics review committees of the London School of Hygiene & Tropical Medicine-United Kingdom and the National Eye Hospital-Sri Lanka. This study adhered to the tenets of the ‘Declaration of Helsinki’ and written informed consent was obtained from all participants. A prospective screening intervention validation study was conducted between May 2017 and May 2018 at a tertiary level, public sector out-patient medical clinic in the Western province of Sri Lanka. The main outcome measure was detection of signs of DR (any DR or a referable level) by physician graders using captured digital images, according to a locally adopted guideline. The protocol of this validation study has been published in Journal of Medical Internet Research (JMIR-doi:10.2196/10900) and a summary is outlined below [24].

### Summary of the methods

Nine general physicians from a tertiary level institution underwent a competency-based training programme following written informed consent, delivered by two retinologists. The training included the following: capturing retinal fields using a hand-held non-mydriatic fundus camera (Zeiss-Visuscout100®-Germany), identification of signs of DR using images and DR grading according to an adopted classification system based on the United Kingdom - National Screening System [25] (Additional file 1: Table S1). The hand-held imaging system has the ability to capture colour and red free retinal images in a range of + 20 diopters (D) to − 20 D, at 40 ^0^ field of view angle. The camera comprised of 9 fixation targets and resolution of the camera is 800 × 480 (5 megapixels). Guidelines were used to standardize reporting of image quality, and ungradable images were classified based on the proportion of the retina visible for grading (Additional file 1: Figure S1). Physicians were tested using a set of standard images of DR and the two who reached the required level of agreement with the retinologist (k = 0.8–0.9) were selected as graders in the validation study.

A sample size of *n* = 506 participants was chosen, in order to estimate the sensitivity within a margin of error 10% (based on 95% confidence intervals), with an expected sensitivity of 70% and prevalence of moderate NPDR among PwDM of 20%. This included an additional 25% to take account of ungradable images (i.e., < 50% of the retina visible). Interim analysis was undertaken to ascertain the level of ungradable images and, to take account of a higher than expected proportion of ungradable images, the sample size was increased to 700 PwDM. A consecutive sample (*n* = 700) of diagnosed PwDM (> 18 years) without previous DRS at an eye clinic who were included in the study following written informed consent. Participants were identified at a tertiary level medical clinic, in the Western province of Sri Lanka.

In the index test imaging, two-field (1st field-macula cantered, 2nd field-disc centred) (Additional file 1: Figure S2), 45-degree retinal images were captured in each eye before and after pupillary dilatation, using 2% phenylephrine, following adequate mydriasis (5–6 mm) by each physician grader. During grading, the non-mydriatic images were graded first. We calculated DTA at 3 levels for the non-ophthalmic settings: i.e., 1) any DR (detection of R1, R2, R3 and R4), 2) referable DR (R2 and above) and 3) detection of referable level and maculopathy combined with a visual acuity cut off (worse eye > 6/18 Snellen visual acuity) (see Additional file 1: Table S1). The graders were masked to the history and clinical examination findings and pupil status of the images. The clinical reference test entailed a detailed, dilated fundus examination by an experienced trainer retinologist using slit-lamp bio-microscopy with a 90D lens and indirect ophthalmoscopy using a 20D lens. The reference test was conducted by one retinologist with more than 15 years of clinical experience in vitreo-retina field. The 7-field ‘Early Treatment diabetic Retinopathy Study’ references test was logistically not feasible in this resource poor setting. This reference examination took place as soon after imaging as possible in all 700 PwDM that were included in the index test. The retinologist was masked to the clinical status and physician graders’ findings.

For quality assurance, 15% of each non-mydriatic and mydriatic image sets were evaluated by the retinologist for technique, ability to image the required field and gradability. Fifteen percent of each hundred image sets were given back to the physician graders for double grading to assess the repeatability and intra-grader agreement in the 1st and 2nd attempts of grading images. A sample of the same image sets (*n* = 212) were graded by the retinologist to calculate inter-grader agreement.

### Analysis

Data were entered in to an MS Excel-16.0 worksheet and transferred to SPSS-Version-20.0 (Armonk-NY-IBM Corp-2011) and STATA/IC-Version-14.2 (Texas-77,845-USA) for analysis. DTA variables of sensitivity, specificity and predictive values and agreement analyses (kappa statistics) were calculated with 95% confidence intervals, for each grader and each pupil status compared to the reference standard using individual eyes as the unit of analysis, considering each gradable eye as a separate case. Two approaches were used in the calculations to examine the impact of ungradable images on the outcomes. i.e., by excluding the ungradable images and by including ungradable as test positive in the analysis. As ungradable images indicate a requirement for referral to an eye clinic, we analysed ungradable images as screen positives to examine the sensitivity and specificity of detecting a need for referral. This also allows comparisons with previous studies, which have used both methods.

Subgroup analysis conducted for identification of presence/absence of DR (any DR), moderate NPDR and above with / without macular signs, to make recommendations for a referable criterion for the local context. We used different referable criteria i.e., by pupil status, level of DR, level of visual acuity and presence of macular signs in the analysis to understand the variation in DTA to assess the most suitable and accurate cut off level of DR without overloading the eye clinic and also facilitating safe practice at a non-ophthalmic setting.

## Results

### Participants’ characteristics

Of the 826 eligible PwDM identified from medical clinical records, response rate was 84.7% (700/826). Mean age of the participants was 60.8 years (SD ±10.08) and majority were women (66%, 462/700). Only 27.9% (195/700) of the participants were employed and 79.1% (554/700) lived in the capital city of Colombo and hailed from low income families (88%, 616/700, monthly income < £150). Of these, 98.4% (689/700) had type 2 DM and 1.6% (11/689) were diagnosed with DM at age < 30 years and were on insulin. The mean age at diagnosis of DM was 50.9 years (SD ±11.03) and mean duration of diabetes was 9.9 years (SD ±8.09). Mean fasting plasma glucose with in the last 3 months was 140.4 mg/dl (SD ±55.43). Additional co-morbidities included; hypertension (70%), hyperlipidaemia (57.3%), ischaemic heart disease (31.9%), nephropathy (9%) and neuropathy (35%). The Table 1 shows the characteristics of the PwDM in this study. The maximum time interval between index and reference test was 4 weeks.

**Table 1:** Participants’ characteristics

Variable	Categories	Results
Mean age	Mean	60.8 years (SD 10.1)
Sex	Male	34% (*n* = 238)
	Female	66% (*n* = 462)
Employment status	Employed	27.9%(*n* = 195)
	Unemployed	41.0% (*n* = 287)
	Retired	31.1%(*n* = 218)
Monthly household income	Low (<£150)	88.0% (*n* = 616)
	Middle (>£150 - < £300)	9.6% (*n* = 67)
	High (>£300)	2.4% (*n* = 17)
Ethnic group	Sinhalese	66.9% (*n* = 468)
	Tamil	16.4% (*n* = 115)
	Moor	14.1%(*n* = 99)
	Other	2.6% (*n* = 18)
Age at diagnosis of diabetes	Mean	50.9 years (SD 11.0)
Duration of diabetes	Mean	9.9 years (SD 8.1)
Current treatment of DM	Diet only	5.6% (*n* = 39)
	Oral medication only	79.7% (*n* = 558)
	Insulin only	5.6% (n = 39)
	Oral medication and insulin	9.1% (*n* = 64)
Fasting glucose level (within 3 months)	Mean	140.44 mg/dl (SD 55.4) 95% CI (136.2–144.0)
HbA1c level (only *n* = 42 available)	Mean	7.9% (SD 2.2) 95% CI (7.3–8.7)
Other comorbidities	Hypertension	70%
	Hypercholesterolaemia	57.3%
	Ischaemic heart disease	31.9%
	Nephropathy	9%
	Neuropathy	35%
	Leg / peripheral ulcers	5%
Age at diagnosis of hypertension	Mean	52.8 years (SD 9.6)
Family history	Diabetes	63.3%
	Hypertension	50.4%
	Hypercholesterolaemia	30%
	Ischaemic heart diseases	28.7%

### Image gradability and number of images sets available for DTA analysis

**Fig. 1:**
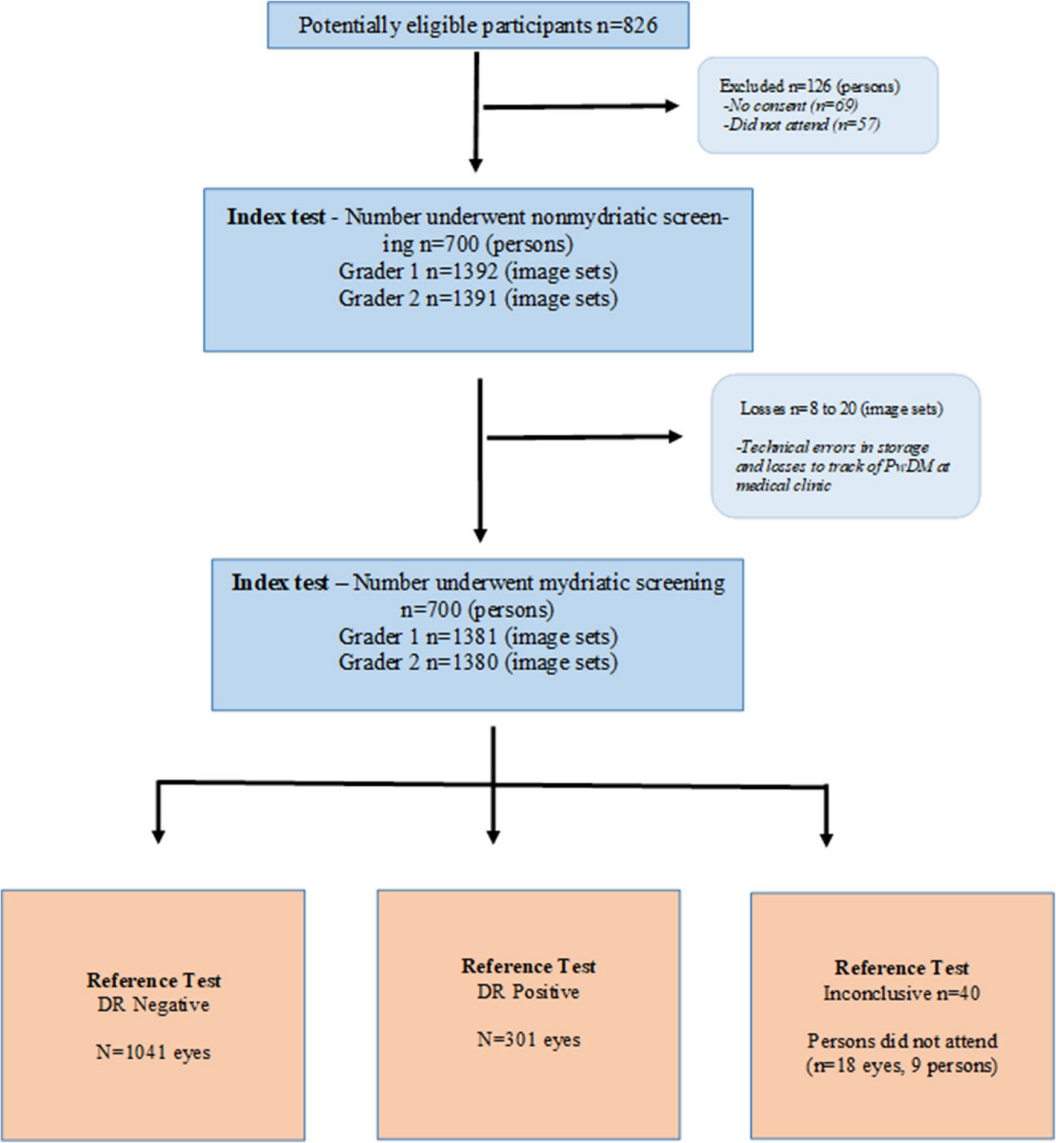
Flow chart of the number of participants and image sets used in the DTA analysis

Seven hundred PwDM were included in the study and 126 (15.2%, 126/826) were excluded (*n* = 69-no consent and *n* = 57-did not attend for imaging) (See Fig. 1). Since both physician graders captured image sets of each participant, ideally there should be 1400 image sets (by eyes) for each grader for each pupil status. However, we ended up as shown in Additional file 2 - flow chart, due to technical errors in storage and failure to track PwDM (8–20 eyes, 0.6–1.4%) at the medical clinic. Overall ungradable proportion in non-mydriatic imaging was 31.0% (217/700) for at least one eye ungradable for either grader. In 12.0% (84/700) both eyes were ungradable for both graders. This decreased to 11.4% (80/700) and 1.1% (8/700) respectively, following pupil dilatation. We noted that 9 PwDM (18 eyes) did not attend for the reference test. In addition, reference test was not possible in 40 eyes (40/1400, 2.8%, in 21 participants: 37 advanced lens opacity, 1 posterior capsular opacity, 1 phthisical eye 1 and 1 eviscerated). After excluding eyes of those who did not attend and ungradable even at the reference test (total *n* = 58) we left with 1342 image sets (by eyes) in DTA analysis. Overall there were 1041 DR positive eyes and 301 DR negative eyes as identified at the reference test. The technical failure rates by the area of visibility of the retinal fields for each image set in the index test by pupil status and grader level (by eyes) are described in the Table 2 and Additional files 2 and 3. In addition, a very good gradability agreement (range k = 0.72–0.96) was observed for physician graders in comparison to retinologist’s findings using a sample of images.

**Table 2:** Gradability of the images as marked by each grader and agreement with the reference grader

Gradability percentage of the retinal fields	Non-mydriatic imaging		Mydriatic imaging	
	Grader 1 *N*^c^ = 1392	Grader 2 *N* = 1391	Grader 1 *N* = 1381	Grader 2 *N* = 1380
Gradable				
100% ^a^	286 (20.5%)	352 (25.3%)	537 (38.9%)	605 (43.8%)
75%	308 (22.1%)	431 (31.0%)	395 (28.6%)	519 (37.6%)
50%	386 (27.7%)	276 (19.8%)	351 (25.4%)	186 (13.5%)
Ungradable				
<50%	412 (29.6%)	332 (23.9%)	98 (7.1%)	70 (5.1%)
Inter-grader Agreement ^b^, kappa (95% CI)	0.90 (0.85,0.95)	0.90 (0.86,0.95)	0.72 (0.56,0.89)	0.96 (0.89,1.03)

^a^. Percentage of visibility in a given field, by eyes

^b^. Physician grader vs retinologist – grading a random sample of image sets (*n* = 212, total *n* = 424)

^c^. Number of image sets by eyes

### DTA after including ungradable images (primary analysis)

We aimed to demonstrate the DTA for referrals to eye clinic rather than the DTA of detecting DR in the primary analysis. When considering the ungradable images as screen positives, sensitivity of detection of any level of DR using non-mydriatic imaging was 82.7% (95% CI 78.4–86.5%) in grader 1 and 78.3% (95% CI 73.7–82.5%) in grader 2. However, since they were referring a higher proportion of ungradable, probably those who did not have the disease, specificity values dropped to 70.4% (95% CI 67.6–73.1%) in grader 1 and 76.2% (95% CI 73.6–78.7%) in grader 2 in non-mydriatic imaging. In mydriatic imaging when we included the ungradable images in the analysis sensitivity was 79.3% (74.7–84.8%) in grader 1 and 78.0% (95% CI 73.4–82.2%) in grader 2. The specificity value of grader 1 was 89.2% (95% CI 87.2–90.9%) and grader 2 was 91.5% (95% CI 89.7–93.1%). The sensitivity, specificity, NPV, PPV and kappa agreement at different levels of DR after including the ungradable images are described in Table 3.

**Table 3:** Diagnostic test accuracy of each grader by each pupil status (unit of analysis, by eyes, after including ungradable images)

Index Test	Sensitivity (95% CI) (%)	Specificity (95% CI) (%)	PPV (95% CI) (%)	NPV (95% CI) (%)	Kappa (95% CI) (%)
Any DR grading					
Non-mydiatric image					
Grader 1	82.7 (78.5, 86.5)	70.4 (67.6, 73.1)	47.4 (43.4, 51.5)	92.7 (90.7, 94.4)	0.42 (0.38, 0.47)
Grader 2	78.3 (73.7, 82.5)	76.2 (73.6, 78.8)	51.6 (47.3, 55.9)	91.6 (89.6, 93.3)	0.46 (0.42, 0.51)
Mydiatric image					
Grader 1	79.3 (74.7, 83.4)	89.2 (87.2, 90.9)	70.3 (65.6, 74.8)	93.0 (91.3, 94.5)	0.66 (0.61, 0.70)
Grader 2	78.0 (73.4, 82.2)	91.5 (89.7, 93.1)	74.7 (70.0, 79.1)	92.8 (91.1, 94.3)	0.68 (0.64, 0.73)
Referable DR grading ^a^					
Non-mydiatric image					
Grader 1	86.8 (79.5, 92.3)	71.7 (69.2, 74.2)	20.4 (16.9, 24.3)	98.5 (97.6, 99.1)	0.23 (0.19, 0.28)
Grader 2	84.9 (77.3, 90.9)	77.3 (75.0, 79.6)	23.8 (19.7, 28.3)	98.4 (97.5, 99.0)	0.29 (0.23, 0.34)
Mydiatric image					
Grader 1	88.7 (81.7, 93.8)	94.9 (93.6, 96.0)	59.1 (51.4, 66.6)	99.0 (98.4, 99.5)	0.68 (0.61, 0.75)
Grader 2	92.5 (86.4, 96.5)	96.4 (95.3, 97.3)	68.0 (60.2, 75.3)	99.4 (98.8, 99.7)	0.76 (0.70, 0.82)
Maculopathy grading ^b^					
Non-mydiatric image					
Grader 1	89.2 (83.5, 93.5)	70.1 (67.5, 72.6)	26.5 (22.7, 30.4)	98.2 (97.2, 98.9)	0.29 (0.25, 0.34)
Grader 2	80.4 (73.5, 86.6)	77.0 (74.6, 79.3)	29.7 (25.3, 34.3)	97.0 (95.8, 98.0)	0.33 (0.28,0.38)
Mydiatric image					
Grader 1	86.5 (80.4, 91.4)	91.5 (89.8, 92.9)	54.9 (48.5, 61.2)	98.3 (97.4, 98.9)	0.62 (0.56,0.68)
Grader 2	82.4 (75.8, 87.9)	95.4 (94.1, 96.5)	68.2 (61.1, 74.7)	97.0 (96.9, 98.6)	0.71 (0.65,0.77)

^a^-Referable level DR – moderate non-proliferative DR and above

^b^-Maculopathy – presence of haemorrhage/s or exudates within 2-disc diameters of the centre of fovea

### DTA after excluding ungradable images

The DTA estimates were calculated after excluding ungradable images (< 50% of the field visible) in the next step as an accuracy measure of the modality. In the comparison physician’s grading using 2-field imaging against the clinical reference standard, in detection of any level of DR, there was no significant difference in DTA by pupil status, in each grader. Similar results were observed in detection of macular signs. Table 4 shows the sensitivity, specificity, positive predictive value (PPV) and negative predictive value (NPV) for each grader and for each pupil status for this analysis. A higher range of PPV values were observed in detecting a referable level DR (79.7–92.8%) (moderate non-proliferative DR and above) compared to identification of macular signs (63.2–73.5%) (presence of haemorrhage/s or exudate/s within 2-disc diameters of centre of fovea). However, such differences were not observed in NPV.

**Table 4:** Diagnostic test accuracy of each grader by each pupil status (unit of analysis, by eyes, after excluding ungradable images)

Index Test	Sensitivity (95% CI) (%)	Specificity (95% CI) (%)	PPV (95% CI) (%)	NPV (95% CI) (%)	Kappa (95% CI) (%)
Any DR grading					
Non-mydiatric image					
Grader 1	71.1 (64.9, 77.4)	95.6 (94.1, 97.0)	80.8 (75.0, 86.6)	92.7 (90.8, 94.5)	0.70 (0.64, 0.76)
Grader 2	66.4 (60.0, 72.7)	95.4 (94.0, 96.8)	78.9 (72.9, 84.9)	91.7 (89.8,93.5)	0.66 (0.60,0.72)
Mydiatric image					
Grader 1	76.2 (71.3, 81.0)	94.0 (92.6, 95.5)	79.1 (74.4, 83.9)	93.0 (91.4, 94.6)	0.71 (0.66, 0.76)
Grader 2	75.2 (70.2, 80.1)	93.9 (92.5, 95.4)	78.3 (73.6, 83.1)	92.9 (91.3, 94.5)	0.70 (0.65, 0.75)
Referable DR grading^a^					
Non-mydiatric image					
Grader 1	73.6 (61.7, 85.5)	99.7 (99.3, 100.0)	92.9 (85.1, 100.7)	98.5 (97.7, 99.3)	0.81 (0.72, 0.90)
Grader 2	71.7 (59.6, 83.8)	99.0 (98.4, 99.6)	79.2 (67.7, 90.7)	98.5 (97.8, 99.3)	0.74 (0.64, 0.84)
Mydiatric image					
Grader 1	81.8 (72.5, 91.1)	99.4 (99.0, 99.9)	88.5 (80.5, 96.5)	99.0 (98.5, 99.6)	0.84 (0.77, 0.91)
Grader 2	89.4 (82.0, 96.8)	98.8 (98.2, 99.4)	79.7 (70.6, 88.9)	99.4 (99.0, 99.9)	0.83 (0.77, 0.90)
Maculopathy grading^b^					
Non-mydiatric image					
Grader 1	78.1 (68.6, 87.6)	96.6 (95.5,97.8)	65.5 (55.5, 75.5)	98.1 (97.29, 99.1)	0.69 (0.60, 0.77)
Grader 2	64.1 (53.5, 74.8)	98.1 (97.3, 99.0)	73.5 (63.0, 84.0)	97.1 (96.1, 98.2)	0.66 (0.57, 0.75)
Mydiatric image					
Grader 1	81.0 (73.3, 88.7)	96.0 (94.9, 97.1)	63.3 (54.9, 71.6)	98.3 (97.6, 99.1)	0.68 (0.61, 0.75)
Grader 2	75.3 (66.8,83.7)	97.8 (96.91, 98.6)	73.8 (65.3,82.3)	97.9 (97.1, 98.7)	0.72 (0.65, 0.79)

^a^-Referable level DR – moderate non-proliferative DR and above

^b^-Maculopathy – presence of haemorrhage/s or exudates within 2 disc diameters of the centre of fovea

### Sub-analyses of DTA

As a pragmatic approach for a resource poor non-ophthalmic setting, we reported the DTA of DRS using non-mydriatic imaging and dilatation of the pupils of only those who have ungradable images (two-step process). In this sub-analysis, the eye which was ungradable even following mydriasis were considered as screen positives. We derived a sensitivity of referable level of DR 81.1% (95% CI 72.9–87.9%) for grader 1 and 82.1% (95% CI 74.0–88.6%) for grader 2. The specificity values were 95.4% (95% CI 94.2–96.5%) for grader 1 and 97.1% (95% CI 96.1–97.9%) for grader 2 in this approach. We observed an improved level of PPV (59.7–70.2%) and NPV (98.4–98.5%) in this strategy. The details are described in Additional file 4.

We combined the DTA of the detection of referable level DR (moderate NPDR and above) with positive macular signs, using non-mydriatic imaging, where the sensitivity was 79.0% for grader 1 and 70.8% for the grader 2. These estimates improved to 84.5 and 85.8% respectively for grader 1 and 2 after dilatation. For the same referable level specificity values were 96.6 and 98.0% for grader 1 and 2 respectively and there was no significant change with the pupil dilatation (non-mydriatic grader 1–97.3%, grader 2–98.4%).

We also incorporated visual acuity threshold for referrals (considering worse eye visual acuity 6/18 and above, retinopathy moderate and above and positive macular signs) and found a sensitivity of grader 1 was 98.3% (95% CI 94.9–99.7%) and grader 2, 97.4% (95% CI 93.5–99.3%). However, in the same referable level specificity values showed an overall reduction (grader-1 49.4, 95% CI 45.3–53.5% and grader-2 51.6, 95% CI 47.5–55.7%), probably due to high number of PwDM referred to the next level without ≥ moderate NPDR. These approaches will be useful in making recommendations for a referable level for a non-ophthalmic setting.

### Agreement analysis

The percentage of image gradability agreement, between index graders and retinologist (inter-grader agreement), in non-mydriatic imaging; grader 1 was 85.2% ([kappa] k = 0.9, 95% CI 0.85–0.95) and grader 2, 78.5% (k = 0.9, 95% CI 0.86–0.95). In mydriatic imaging, inter-grader gradability agreement of grader 1 was 76.2% (k = 0.72, 95% CI 0.56–0.89) and grader 2, 72.7% (k = 0.96, 95% CI 0.89–1.03).

We proposed grading the same images by the retinologist would provide a fair comparison for the physician graders in agreement analysis. However, here the concerns were limitations in the degree of view of a hand-held retinal camera and skills of capturing images by the physicians. In this analysis, in the grading of DR and macular signs, we found that inter-grader agreement was mostly > 0.8 except for the grader 2 non-mydriatic images (any DR k = 0.80–0.89, referable DR k = 0.77–0.85, macular signs k = 0.77–0.85). We observed a satisfactory level of agreement of the physician graders findings using a 2-field modality. The overall concordance of the results is described in Table 5.

**Table 5:** Agreement by comparing findings of sample of same images (captured by physicians) graded by retinologist (inter-grader agreement: physician grader 1 or 2 vs retinologist) (*n* = 212, 424 image sets)

Index Test	Sensitivity (95% CI) (%)	Specificity (95% CI) (%)	Kappa value (95% CI) (k)
Any DR grading			
Non-mydriatic image			
Grader 1	92.3 (86.4, 98.2)	96.8 (94.5, 99.1)	0.89 (0.83, 0.95)
Grader 2	84.9 (77.3, 92.5)	94.9 (92.1, 97.7)	0.80 (0.73, 0.88)
Mydriatic image			
Grader 1	90.2 (85.0, 95.5)	96.7 (94.6, 98.8)	0.88 (0.82, 0.93)
Grader 2	90.5 (85.5, 95.6)	95.0 (92.5, 97.6)	0.85 (0.80, 0.91)
Referable DR grading ^a^			
Non-mydriatic image			
Grader 1	80.0 (64.3, 95.7)	99.3 (98.3, 100.3)	0.84 (0.72, 0.96)
Grader 2	79.2 (62.3, 95.4)	98.3 (96.9, 99.8)	0.77 (0.64, 0.91)
Mydriatic image			
Grader 1	77.1 (63.2, 91.1)	98.9 (97.8, 100.0)	0.80 (0.69, 0.91)
Grader 2	97.0 (91.1, 102.9)	97.6 (96.4, 99.2)	0.85 (0.76, 0.94)
Maculopathy grading ^b^			
Non-mydriatic image			
Grader 1	94.3 (86.6, 102.0)	97.0 (94.9, 99.0)	0.85 (0.76, 0.94)
Grader 2	75.0 (61.6, 88.5)	98.2 (96.7, 99.8)	0.77 (0.66, 0.88)
Mydriatic image			
Grader 1	88.1 (79.9, 96.4)	96.5 (94.5, 98.4)	0.82 (0.74, 0.90)
Grader 2	76.6 (66.2, 86.9)	98.5 (97.3, 99.8)	0.80 (0.72, 0.89)

^a^-Referable level DR – moderate non-proliferative DR and above

^b^-Maculopathy – presence of haemorrhage/s or exudates within 2-disc diameters of the centre of fovea

### Quality assurance

The index graders re-graded the coded image sets in a masked fashion independently without having access to the first attempt data. In this, first attempt vs second attempt weighted kappa agreement was calculated to assess the repeatability of DR grading at level of retinopathy. The kappa value of grader 1 was 0.69 (95% CI 0.60–0.78) and grader 2, 0.66 (95% CI 0.58–0.73). In comparison of grader 1 vs grader 2, first attempt kappa was 0.82 (95% CI 0.76–0.89) and in second attempt it was 0.74 (95% CI 0.66–0.83%) (see Additional file 5).

### Reasons for ungradability of images, prevalence of DR and time and flow of the participants

We described the possible reasons for ungradability, using the highest recorded ungradability values, which was observed by grader 1. Of the 29.4% of ungradable images for grader 1, non-mydriatic imaging, 69.2% (285/412) had lens opacity. Among these 29.8% (85/285) eyes had significant level of lens opacity which required cataract assessment. Following reference test 37 eyes were identified as having lens opacities that required urgent cataract surgery. Overall, 75.6% of the participants had no DR, 16.7% mild DR (R1), 3.6% moderate NPDR (R2), 0.4% severe NPDR (R3) and only 1% had PDR (R4). Among the ungradable images in non-mydriatic imaging, 66.5% (274/412) had no retinopathy (R0), 19.9%-mild NPDR (R1), 1.7%-moderate NPDR (R2), 0.7%-severe NPDR (R3) and 1.5%-proliferative DR (PDR-R4) (see Additional file 3).

The mean time gap between index imaging and reference test was 3.6 days (SD ± 0.2) (95% CI 3.2–4.0, range 0–48 days). Six hundred and ninety-two PwDM completed the reference test examination and 98% (684/692) of them underwent retinologists examination < 4 weeks period.

## Discussion

We demonstrated that DRS by general physicians using a mydriatic two field technique was a feasible modality to detect a defined level of referable DR (moderate NPDR and above, after including ungradable images: sensitivity 88.7–92.5% and specificity 94.9–96.4%) in a non-ophthalmic setting, considering the level of DTA achieved. This may be suitable for LMIC settings where it would be difficult to implement full population based DRSP due to resource and information constraints. Compared to a locally accepted clinical reference standard, DRS using mydriatic 2-field strategy by general physicians showed an accepted level of DTA which most of the HIC screening programs follow (sensitivity of > 80% and specificity of > 95%) [25]. We assumed that inclusion of ungradable images in the DTA analysis is a pragmatic approach for a non-ophthalmic setting, considering the requirement of referring those PwDM to the eye clinic for further assessment and treatment. The proposed imaging strategy could act as a filter minimizing the number of referrals at eye clinic, thereby reducing the strain on the system. The United Kingdom prospective diabetes study group (UKPDS) reported that 15.3% of those with signs of DR at baseline, required laser at 3 years [26]. Therefore, identification of even minor levels of DR will be beneficial to stratify the risk groups early.

Digital retinal imaging showed promising results in DRS [25]. The digital imaging systems have the advantage of instant availability of images for quality assessment and convenient storage and retrieval. Several studies have compared digital fundus photography with 7-fields used in early treatment diabetic retinopathy study (ETDRS) [27–29] or mydriatic ophthalmoscopy [30, 31] in DRS and shown an acceptable level of DTA. The DTA studies from high income countries (HIC) used trained graders or ophthalmologists/retinologists in index test and table top static cameras with advanced technology such as wider angle and high resolution, which may be prohibitively expensive for LMICs. Though DTA is lower in this study, this strategy would be useful in a context where there is no systematic DRS. On the other hand, it may be arbitrary to compare the findings of this study with HICs. LMICs such as Sri Lanka require pragmatic solutions for control of visual loss due to DR with rising prevalence of DM.

The optimum number of retinal fields in a DRS strategy is a key factor that affects accuracy. The ETDRS 7-field strategy is considered to be the gold standard for DR detection but is not practical in a screening program [32]. Previous studies showed that single retinal field is inadequate to achieve required standards [33–37]. Studies have also demonstrated that 3-fields would not improve DTA of detection of any referable DR [38]. A non-mydriatic two field strategy in detection of sight threatening DR (STDR) in a HIC showed a sensitivity of 92% (95% CI 90–94%) and specificity of 96% (95% CI 95–98%) (proportion of ungradability - non-mydriatic 15.3–17.6%, mydriatic 1.4–2.1%) [27]. In our study, sensitivity was 71.7–73.5% and specificity 98.9–99.6% for detection of referable DR using non-mydriatic imaging. We could not achieve a higher level of sensitivity comparable with the studies done in HICs, due to poor image quality. The main causes of poor image quality are dark iris colour, poor pupil dilation status and lens opacity [17, 39]. In HICs prevalence of cataract is less compared to LMICs like Sri Lanka [40–42]. We observed that sensitivity increased to 81.8–89.3% when pupils were dilated. In addition, specificity was high irrespective of the pupil status, because physician graders were confident in grading in the absence of any signs. The study by Henricsson, M. et al. (2000) showed that dilatation and increasing number of fields to 5, the DTA improved to sensitivity of 93% and specificity of 91% [43]. It is apparent that one or more fields to the two central fields in DRS, has increased DTA minimally [28]. Therefore, a 2-field DRS strategy is justifiable for this context. In addition, slit-lamp examination by the retinologist is a justifiable reference test for this context. Scanlon, PH. et al., (2003) showed that there was no significant difference in the assessment of DTA between using 7-field ETDRS and slit lamp examination by ophthalmologists [31].

In some settings, several non-ophthalmological personnel had been employed in DRS. In our study we proposed DRS by trained general physician at medical clinic following assessment of barriers. The estimates from previous studies are comparable with the finding of our study [39]. In a study from the United Kingdom, DRS by general practitioners using 35 mm colour images shown that detecting any level of DR was increased from 62.6% (95% CI 55.9–69.4%) with direct ophthalmoscopy to 79.2% (95% CI 73.6–84.9%) using retinal photographs (and specificity remained unchanged (direct ophthalmoscopy 75.0% (95% CI 69.5–80.5%) vs 73.5% (95% CI 68.0–79.1%)) [44]. They concluded that retinal photography by trained general practitioners in primary care setting could achieve an acceptable level of detection of STDR (87%) [44]. In our validation study physician graders showed a sensitivity range of 88.6–92.4% and specificity range of 94.8–96.3% in detection of referable level of DR using mydriatic imaging which is better than the reported studies. However, this may depend on the proportion of ungradable images. In our sub-analysis we included the technical failures as test positives, since physician graders refer these to the eye clinic. A review of 22 cross sectional photographic studies showed non-mydriatic retinal photography sensitivity range of 25–66% for general practitioners, 43–79% for optometrists and 27–73% for other non-ophthalmic health professionals and an overall specificity of > 91% [45]. The sensitivity of detection of any level of DR increased to 87–100% for general practitioners and > 91% for optometrists with pupil dilatation [45]. As a first line, this study has shown that physician graders are capable of DRS in a non-ophthalmic setting in Sri Lanka. However, we will have to study the effectiveness of this modality in a larger number to make specific recommendations to implement a population-based program.

In our study 75.6% of the participants had no DR, 16.7% mild DR (R1), 3.6% moderate NPDR (R2), 0.4% severe NPDR (R3) and only 1% had PDR (R4). A study conducted among the slum populations (age > 40 years, known PwDM) in India, using a hand-held nonmydriatic camera reported 8.1% severe NPDR and 6.8% PDR which are higher prevalence than our study [46]. One reason for higher prevalence could be poor diabetes management among the slum populations. However, in this study, relatively a higher gradability of images (89.4% gradable) was observed even in non-mydriatic mode, probably due to images were graded at the site after directly visualising on the display of the hand-held camera. We have noticed that image quality is apparently higher on a small screen compared to displaying on a traditional viewing monitor. In another study conducted in India, among 500 PwDM at an endocrinology clinic, proportion ungradable was 30.6 and 31% among two observers which is comparable to our results [39]. In comparison we observed that studies conducted in HICs reported high proportions of gradability compared to our study. A study conducted in USA 86–94% images were gradable before pupil dilatation in hand-held retinal imaging [47]. Similarly a study conducted in a upper middle income setting (China) reported a low ungradable proportion of 4.75% (19/400) using a hand-held camera [21]. The low prevalence of DR in our study could be attributed to many factors. One reason for this would be excluding those who had undergone previous DRS and treatment. In Sri Lanka about 50% of the PwDM in clinics had DRS [48]. Forty percent (572/1398) of the PwDM had previous DRS or DR treatment in our study. The high proportion poor image quality in our study could be due to smaller pupil size and presence of lens opacities.

Non-mydriatic imaging has lower resolution and lower image quality leading to poorer detection of DR [17, 49]. However, digital imaging has lower technical failure rates than imaging using colour slides [50]. The hand-held non-mydriatic camera used in this study required a minimum of 3.5 mm pupil diameter and average pupil diameter in this study population was 2.01 mm at presentation. When pupils were dilated, proportion of ungradable images was reduced from 43.4 to 12.8%. Even at the reference test 37 eyes were ungradable due to lens opacity. The improvement of image quality in people with dark irises by pupil dilatation has been demonstrated in a previous study [17]. The referral of ungradable images to an ophthalmologist’s clinic is in the best interest of patient safety. Scanlon, P. et al. showed that in the > 80 years age group the technical failure rates reduced from 41.6 to 16.9% following mydriasis [18]. This study concluded that the odds of having one eye ungradable, increased by 2.6% (95% CI 1.6–3.7%) for each extra year of life since diagnosis of DM and major cause of ungradable images was having a central cataract (57%) [18]. Therefore, a non-mydriatic strategy with dilatation of pupil for ungradable images only would be more appropriate for this context. Another reason for low DTA in non-mydriatic imaging could be low resolution, which may have led to poor visibility of delicate signs such as microaneurysms, as suggested in the study by Henriccson et al. (2000) [43].

### Limitations

We excluded PwDM with previous eye screening or treatment, which reduced the proportion of people with DR, which may have introduced spectrum bias. However, the resulting sample included a wide range of pathologies, albeit with fewer people with more advanced disease. When considering any DR as referable level, there were 301 DR positive and 1041 DR negative eyes in the analysis. There were only 69 DR positive eyes when considering moderate NPDR and above as the referable level. However, PwDM who already visited the eye clinic would not usually participate in a screening programme, therefore the sample examined in this study reflects the PwDM who would be eligible for DRS. Another limitation was high proportion of ungradable images from non-mydriatic imaging compared to other studies. However, the populations in LMICs have a higher prevalence of untreated cataracts, which would prevent adequate retinal view and would require referral. In a potential DRSP, these participants will be referred to the next level of eye care and the patient would benefit from the imaging even if the DR status remains unknown.

The most common gold standard for a reference test would be the ETDRS 7-field image grading by an expert grader. However, it was not possible in this setting for a large sample due to resource and time limitations. In addition, any misclassifications in the clinical reference test could have been mitigated, with a second reference grader. In order to have a higher precision of the DTA, the sample size should be adjusted according to the reported low prevalence of higher grades of DR such as severe NPDR and PDR.

Our proposed DRS modality of using a hand-held non-mydriatic retinal camera at a medical clinic may be more appropriate for a resource poor LMIC setting, with the rising prevalence of DM. However, the caution is quality of the images. Our findings may not applicable to a HIC setting where there are more resources and avenues for development of a population-based DRS program using table-top digital imaging systems. On the other hand, this modality can be piggy back in a population-based program in any setting, to improve the access.

## Conclusion

In this study we demonstrated that the diagnostic test accuracy of the physician graders was closer to the standard practice of national level screening programs in other settings. We conclude that 2-field retinal imaging using a hand-held digital camera at a medical clinic, by physician graders, with dilatation of pupil of those who have ungradable images, provides a valid modality to identify referable level of diabetic retinopathy. This strategy is an accurate screening method of detection of a referable level in a health care facility-based people with diabetes who are at risk of developing sight threatening diabetic retinopathy.

## Additional files

Additional file 1: Image quality evaluation and diabetic retinopathy grading classification system. **Figure S1.** Evaluation of image quality. **Figure S2.** Two retinal images captured. **Table S1.** Diabetic retinopathy classification system (DOCX 989 kb)

Additional file 2: Detailed flow chart of the number of participants and image sets used in the analysis (DOCX 57 kb)

Additional file 3: Prevalence of lens opacity and other condition that would affect image gradability and reference test examination (DOCX 16 kb)

Additional file 4: Diagnostic test accuracy for two step grading process (DOCX 14 kb)

Additional file 5: Intra-grader agreement analysis of double grading (DOCX 13 kb)

## Abbreviations

D: Diopters
DM: Diabetes mellitus
DR: Diabetic retinopathy
DRS: Diabetic retinopathy screening
DRSP: Diabetic retinopathy screening program
DTA: Diagnostic test accuracy
ETDRS: Early treatment diabetic retinopathy study
HIC: High income countries
IDF: International diabetes federation
LMIC: Low and middle income countries
NPDR: Non-proliferative diabetic retinopathy
NPV: Negative predictive value
PDR: Proliferative diabetic retinopathy
PPV: Positive predictive value
PwDM: People with diabetes mellitus
STDR: Sight threatening diabetic retinopathy
