# Supplementary material for: Diagnostic test accuracy of diabetic retinopathy screening by physician graders using a hand-held non-mydriatic retinal camera at a tertiary level medical clinic
Source: BMC Ophthalmol. 2019 Apr 8;19:89. doi: 10.1186/s12886-019-1092-3 (PMC6454614; doi:10.1186/s12886-019-1092-3)
Supplement: Supplementary file 1 — Image quality evaluation and diabetic retinopathy grading classification system. Figure S1. Evaluation of image quality. Figure S2. Two retinal images captured. Table S1. Diabetic retinopathy classification system (DOCX 989 kb) [file 12886_2019_1092_MOESM1_ESM.docx]

**Additional File 1.**

**Figure 1. Evaluation of image quality - levels of gradability based on the proportion of the image which can be graded**


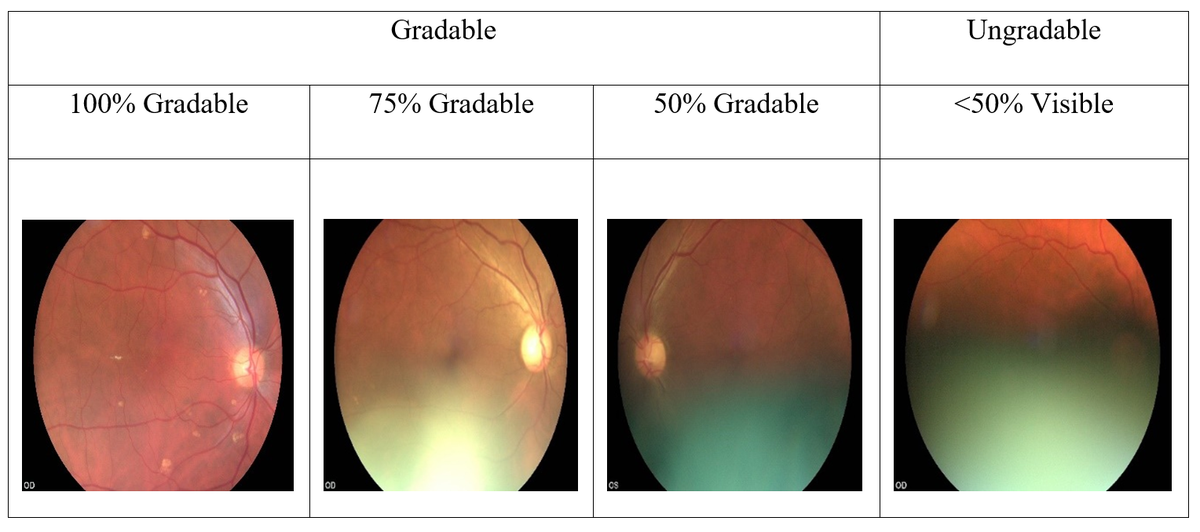


**Additional File 1 - Figure 2. Two retinal images captured**


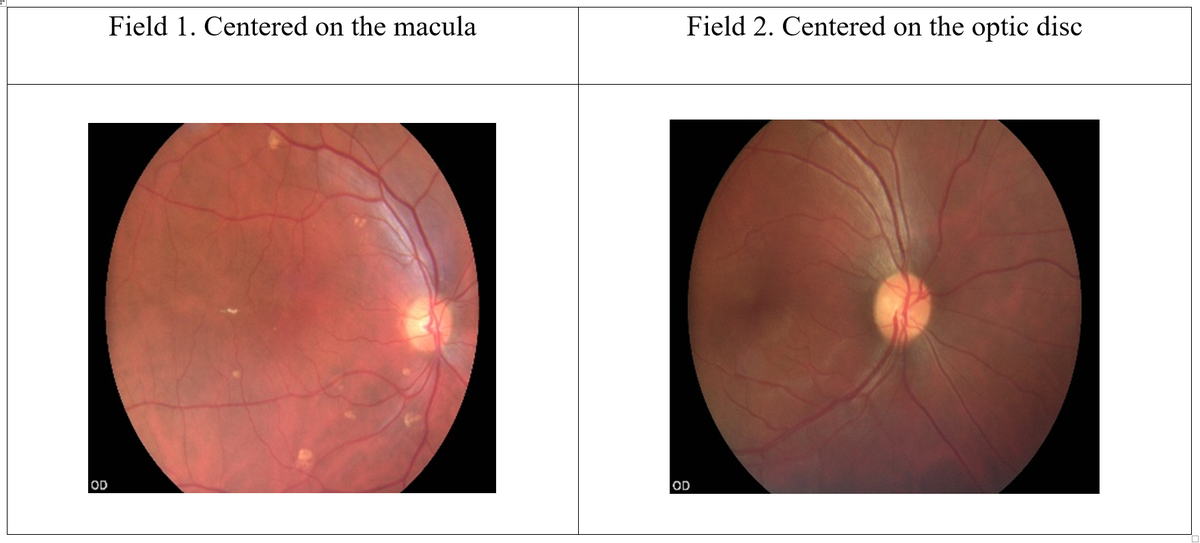


**Additional File 1. DR Classification system**

**Table 1.** Adapted diabetic retinopathy classification for the validation study

| **Signs** | **No DR (R0)** | **Mild BDR^d^ / NPDR^e^**  **(R1)** | **Moderate BDR / NPDR (R2)** | **Severe NPDR**  **(R3)** | **Proliferative DR (PDR^f^) (R4)** |
| --- | --- | --- | --- | --- | --- |
| Microaneurysms | No | Few | Multiple | Multiple | Present |
| Hard Exudates ^a^ | No | Few | Multiple | Multiple | Present |
| Cotton wool spots | No | Occasional | Multiple | Multiple | Present |
| Intra retinal haemorrhage ^a^ | No | Few | >20 in 1-3 quadrants | >20 in 4 quadrants | Present |
| Venous beading | No | Occasional | Present in 1-2 quadrants | Present in >2 quadrants | Present |
| IRMA ^b^ | No | No | Present ~1 quadrant | Prominent >1 quadrant | Present |
| NVD ^c^ | No | No | No | No | Present |
| NVE ^c^ | No | No | No | No | Present |
| Vitreous / pre-retinal haemorrhage | No | No | No | No | Present - advanced PDR |
| Traction | No | No | No | No | Present - advanced PDR |
| Fibrosis | No | No | No | No | Present - advanced PDR |

^a^ Not within the definition of maculopathy

^b^ Intra retinal microvascular abnormalities

^c^ Neo-vascularisations over the disc / elsewhere

^d^ Background DR, ^e^ NPDR – Non-proliferative DR, ^f^ PDR-Proliferative DR

**Table 2.** Macular signs classification

|  | **Maculopathy absent**  **(M0)** | **Maculopathy present**  **(M1)** |
| --- | --- | --- |
| Signs up to 2-disc diameters from the centre of fovea | No signs | Presence of hard exudate/s and / or blot haemorrhage/s  (Referable) |
