# Supplementary material for: Diagnostic test accuracy of diabetic retinopathy screening by physician graders using a hand-held non-mydriatic retinal camera at a tertiary level medical clinic
Source: BMC Ophthalmol. 2019 Apr 8;19:89. doi: 10.1186/s12886-019-1092-3 (PMC6454614; doi:10.1186/s12886-019-1092-3)
Supplement: Supplementary file 2 — Detailed flow chart of the number of participants and image sets used in the analysis (DOCX 57 kb) [file 12886_2019_1092_MOESM2_ESM.docx]

**Additional File 2. Detailed flow chart of the number of participants and image sets used in the analysis**

(Gr 1-Grader 1, Gr2-Grader 2, Nonmyd-Nonmydriatic, Myd-Mydriatic, DNA-did not attend, ^for any DR, DR+ any DR positive, DR- no DR, *I-Inconclusive) (According to the STARD guidelines)

Potentially eligible participants n=826

n=

Excluded n=126 (persons)

*-No consent (n=69)*

*-Did not attend (n=57)*

**Reference standard**

Gr 1 Nonmyd n=790

Gr 2 Nonmyd n=865

Gr1 Myd n=998

Gr 2 Myd n=1026

**Final diagnosis**

Gr 1 Nonmyd (DR+ 143, DR- 34)

Gr 2 Nonmyd (DR+ 142, DR- 38)

Gr1 Myd (DR+ 224, DR- 59)

Gr 2 Myd (DR+ 221, DR- 61)

**Final diagnosis**

Gr 1 Nonmyd (^DR+ 58, DR- 732)

Gr2 Nonmyd (DR+ 72, DR- 792)

Gr1 Myd (DR+ 70, DR- 928)

Gr 2 Myd (DR+ 73, DR- 952)

*-*

**Final diagnosis**

Gr 1 Nonmyd (DR+ 98, DR- 274, *I-37)

Gr 2 Nonmyd (DR+ 85, DR- 209, I-37)

Gr 1 Myd (DR+ 7, DR- 54, I-37)

Gr 2 Myd (DR+ 6, DR- 28, I -37)

**Reference standard**

Gr 1 Nonmyd n=409

Gr 2 Nonmyd n=330

Gr 1 Myd n=98

Gr 2 Myd n=70

**Reference standard**

Gr 1 Nonmyd n=177

Gr 2 Nonmyd n=180

Gr1 Myd n=283

Gr 2 Myd n=281

No reference test n=2-3 (image sets)

*-DNA*

n=12

*-Reason 1 (n=)*

*-Reason 2 (n=)*

No reference test n=1-2 (image sets)

*-DNA*

n=12

*-Reason 1 (n=)*

*-Reason 2 (n=)*

No reference test n=2-12 (image sets)

*-DNA*

n=12

*-Reason 1 (n=)*

*-Reason 2 (n=)*

**Index test - Inconclusive**

Gr 1 Nonmyd n=412

Gr 2 Nonmyd n=332

Gr 1 Myd n=98

Gr 2 Myd n=70

**Index test - Positive**

Gr 1 Nonmyd n=178

Gr 2 Nonmyd n=182

Gr 1 Myd n=283

Gr 2 Myd n=282

**Index test - Negative**

Gr1 Nonmyd n=802

Gr 2 NonMyd n=877

Gr1 Myd n=1000

Gr 2 Myd n=1028

**Index test** - Number underwent nonmydriatic screening n=700 (persons)

Grader 1 n=1392 (image sets)

Grader 2 n=1391 (image sets)

Losses n=8-20 (image sets)

*-Technical errors in storage or losses track PwDM*

**Index test** – Number underwent mydriatic screening n=700 (persons)

Grader 1 n=1381 (image sets)

Grader 2 n=1380 (image sets)
