# Supplementary material for: Diagnostic test accuracy of diabetic retinopathy screening by physician graders using a hand-held non-mydriatic retinal camera at a tertiary level medical clinic
Source: BMC Ophthalmol. 2019 Apr 8;19:89. doi: 10.1186/s12886-019-1092-3 (PMC6454614; doi:10.1186/s12886-019-1092-3)
Supplement: Supplementary file 3 — Prevalence of lens opacity and other condition that would affect image gradability and reference test examination (DOCX 16 kb) [file 12886_2019_1092_MOESM3_ESM.docx]

**Additional File 3.**

**Table 1. Prevalence of lens opacity and other condition that would affect image gradability and reference test examination**

|  | **Nonmydriatic imaging** | **Mydriatic imaging** | **Reference test -Mydriatic** | **Prevalence of DR and Macular signs among ungradable** |
| --- | --- | --- | --- | --- |
|  | **Grader 1*** | **Grader 1** |  |  |
|  |  |  |  |  |
| **Number gradable** | 980 (70%) | 1283 (91.6%) | 1342 (95.8%) |  |
| **Number ungradable** | 412 (29.4%) | 98 (7.0%) | 40 (2.8%) |  |
| **Missing image files or data** | 8 (0.6%) | 19 (1.4%) | 18 (1.3%)  (did not attend for reference test) |  |
|  |  |  |  |  |
| **Lens status** | Lens opacity 285 (69.2%)  Nuclear opalescence  NO 1 – 41  NO 2 – 60  NO 3 – 108  NO 4 – 40  NO 5 – 14  NO 6 – 8  Mature 12  Congenital 2  Posterior subcapsular opacity 218  P1 – 21  P2 – 22  P3 – 15  P4 – 5  P 5 – 4  Cortical cataract  C1 – 4  C2 – 7  C3 – 2 | Lens opacity 78 (79.6%)  Nuclear opalescence  NO 1 – 4  NO 2 – 5  NO 3 – 24  NO 4 – 16  NO 5 – 9  NO 6 – 8  Mature – 12  Posterior subcapsular opacity 58  P1 – 3  P2 – 3  P 3 – 6  P 4 – 4  P 5 – 4  Cortical cataract  C2 - 1 | Lens opacity 37 (92.5%)  Nuclear opalescence  NO 6 only – 10  NO 5 only – 3  NO 4 only – 4  Other combinations  NO5 and P2 – 2  NO4 and P4 – 4  NO 4 and P 3 – 1  NO 3 and P3 – 1  Mature cataract 12 | Prevalence of DR among the ungradable images (non-mydriatic)  R0 274 66.5%  R1 82 19.9%  R2 7 1.7%  R3 3 0.7%  R4 6 1.5%  R9 37 9.0%  Missing 3 0.7%  Prevalence of maculopathy among the ungradable images in nonmydriatic  M0 336 81.6%  M1 29 7.0%  M9 44 10.7%  Total 409 99.3%  Missing 3 0.7% |
|  | Lens clear 122 (29.6%)  clear phakic 40  Aphakic 2 Pseudophakik 80 | Lens clear 15 (15.3%)  Clear phakic 5  Pesudophakic 10 | N/A | Prevalence of DR among the ungradable images (mydriatic)  R0 54 55.1  R1 4 4.1  R4 3 3.1  R9 37 37.8  Prevalence of maculopathy among the ungradable images in mydriatic  M0 57 58.2  M1 1 1.0  M9 40 40.8 |
|  |  |  |  |  |
| **Posterior capsule status** | PCO+  20 | PCO+  3 | PCO +  1 |  |
|  |  |  |  |  |
| **Corneal status** | Corneal opacity 02 (minor) | No | No |  |
|  |  |  |  |  |
| **Other conditions** | Phthysical 1 | Phthysical 1 | Phthysical 1 |  |
|  | Eviscerated 1 | Eviscerated 1 | Eviscerated 1 |  |
|  |  |  |  |  |
| **Mean pupil diameter** | 2.01 mm  SE 0.004  (95% CI 2.007 – 2.026) mm | 6.023 mm  SE 0.032  (95% CI 5.96 – 6.08) mm |  |  |
|  |  |  |  |  |

*The image set of highest ungradability proportion recorded by the grader 1 considered here.
