# Supplementary material for: Diagnostic test accuracy of diabetic retinopathy screening by physician graders using a hand-held non-mydriatic retinal camera at a tertiary level medical clinic
Source: BMC Ophthalmol. 2019 Apr 8;19:89. doi: 10.1186/s12886-019-1092-3 (PMC6454614; doi:10.1186/s12886-019-1092-3)
Supplement: Supplementary file 4 — Diagnostic test accuracy for two step grading process (DOCX 14 kb) [file 12886_2019_1092_MOESM4_ESM.docx]

**Additional File 4.**

**Table 1. DTA for two step grading process - (DTA for gradable nonmydriatic images and nonmydriatic ungradable eyes classified based on mydriatic grading)**

| **Index Test** | | **Sensitivity (95% CI) (%)** | **Specificity  (95% CI) (%)** | **PPV  (95% CI) (%)** | **NPV  (95% CI) (%)** | **Kappa  (95% CI)**  **(%)** |
| --- | --- | --- | --- | --- | --- | --- |
| **Any DR grading** | |  | | | | |
| Two Step Grading * | |  | | | | |
|  | Grader 1 | 75.6  (70.8, 79.98) | 90.8  (85.6, 92.4) | 72.5  (67.7, 77.1) | 92.0  (90.2, 93.6) | 0.65  (0.61, 0.70) |
|  | Grader 2 | 72.5  (67.6, 77.1) | 93.5  (91.8, 94.8) | 78.1  (73.3, 82.5) | 91.4  (89.6, 92.9) | 0.68  (0.63, 0.72) |
| **Referable DR grading** ^^^ | |  | | | | |
| Two Step Grading | |  | | | | |
|  | Grader 1 | 81.1  (72.9, 87.8) | 95.4  (94.2, 96.5) | 59.7  (51.6, 67.5) | 98.4  (97.6, 98.9) | 0.66  (0.59, 0.73) |
|  | Grader 2 | 82.1  (74.0, 88.6) | 97.1  (96.1, 97.9) | 70.2  (61.8, 77.7) | 98.5  (97.7, 99.1) | 0.73  (0.67, 0.80) |

*All non-mydriatic imaging ungradable eyes were replaced by mydriatic grading. The ungradable images even after dilating pupils considered as screen positive.

^ Moderate NPDR and above. Maculopathy not considered.
