# Supplementary material for: Diagnostic test accuracy of diabetic retinopathy screening by physician graders using a hand-held non-mydriatic retinal camera at a tertiary level medical clinic
Source: BMC Ophthalmol. 2019 Apr 8;19:89. doi: 10.1186/s12886-019-1092-3 (PMC6454614; doi:10.1186/s12886-019-1092-3)
Supplement: Supplementary file 5 — Intra-grader agreement analysis of double grading (DOCX 13 kb) [file 12886_2019_1092_MOESM5_ESM.docx]

**Additional File 5.**

**Table 1. Intra-grader agreement analysis of double grading**

(sample size 15% of each 100 image sets)

|  | **Grader 1 vs Grader 1**  **(1^st^ vs 2^nd^)** | **Grader 1 vs Grader 2**  **(1^st^ attempt)** | **Grader 2 vs Grader 2**  **(1^st^ vs 2^nd)^** | **Grader 2 vs Grader 1**  **(2^nd^ attempt)** |
| --- | --- | --- | --- | --- |
| **Binary gradability of images**  **(irrespective of the pupil status)**  **(kappa) (95% CI)** | 0.48  (0.34,0.62) | 0.83  (0.71, 0.95) | 0.85  (0.72, 0.97) | 0.51  (0.37, 0.64) |
| **Retinopathy grading agreement**  **(at each level of R1, 2, 3 and 4; Weighted linear kappa) (k) (95% CI)** | 0.69  (0.60, 0.78) | 0.82  (0.76, 0.89) | 0.66  (0.58, 0.73) | 0.74  (0.66, 0.83) |
| **Agreement of detection of macular signs**  **(k) (95% CI)** | 0.58  (0.45, 0.72) | 0.75  (0.65, 0.85) | 0.71  (0.59, 0.82) | 0.75  (0.65, 0.85) |
